# What implementation strategies and outcome measures are used to transform healthcare organizations into learning health systems? A mixed-methods review protocol

**DOI:** 10.1186/s12961-022-00898-z

**Published:** 2022-09-06

**Authors:** Mari Somerville, Christine Cassidy, Janet Curran, Melissa Rothfus, Doug Sinclair, Annette Elliott Rose

**Affiliations:** 1 IWK Health, Halifax, NS Canada; 2 School of Nursing, Faculty of Health, Dalhousie University, Halifax, NS Canada; 3 W.K. Kellogg Health Sciences Library, Dalhousie University, Halifax, NS Canada

**Keywords:** Learning health system, Health services, Systematic review

## Abstract

**Background:**

A learning health system (LHS) framework provides an opportunity for health system restructuring to provide value-based healthcare. However, there is little evidence showing how to effectively implement a LHS in practice.

**Objective:**

A mixed-methods review is proposed to identify and synthesize the existing evidence on effective implementation strategies and outcomes of LHS in an international context.

**Methods:**

A mixed-methods systematic review will be conducted following methodological guidance from Joanna Briggs Institute (JBI) and PRISMA reporting guidelines. Six databases (CINAHL, Embase, MEDLINE, PAIS, Scopus and Nursing & Allied Health Database) will be searched for terms related to LHS, implementation and evaluation measures. Three reviewers will independently screen the titles, abstracts and full texts of retrieved articles. Studies will be included if they report on the implementation of a LHS in any healthcare setting. Qualitative, quantitative or mixed-methods study designs will be considered for inclusion. No restrictions will be placed on language or date of publication. Grey literature will be considered for inclusion but reviews and protocol papers will be excluded. Data will be extracted from included studies using a standardized extraction form. One reviewer will extract all data and a second will verify. Critical appraisal of all included studies will be conducted by two reviewers. A convergent integration approach to data synthesis will be used, where qualitative and quantitative data will be synthesized separately and then integrated to present overarching findings. Data will be presented in tables and narratively.

**Conclusion:**

This review will address a gap in the literature related to implementation of LHS. The findings from this review will provide researchers with a better understanding of how to design and implement LHS interventions. This systematic review was registered in PROSPERO (CRD42022293348).

**Supplementary Information:**

The online version contains supplementary material available at 10.1186/s12961-022-00898-z.

## Introduction

Value-based healthcare is recognized as a global health priority [1, 2]. This includes care that is patient-centred, evidence-based, cost-efficient and focused on improving health outcomes [3–5]. High-quality care is arguably the most important part of value, but it should be delivered at the optimal cost while ensuring it meets patient-identified outcomes and expectations [5]. Value-based healthcare has not yet been universally adopted, and there is a need to support health systems to achieve this goal. Evidence suggests that providing high-quality care can reduce unnecessary deaths and medication errors [2]. Further, value-based care can lower healthcare costs by focusing on quality of care per service rather than number of healthcare interactions [4, 6]. Value-based healthcare is clearly beneficial for society, yet health systems face challenges in achieving optimal, quality care.

A learning health system (LHS) has been identified as a promising approach for enabling value-based healthcare at a systems level. The Institute of Medicine in 2007 defined LHS as a system in which “science, informatics, incentives, and culture are aligned for continuous improvement and innovation, with best practices seamlessly embedded in the delivery process and new knowledge captured as an integral by-product of the delivery experience” [7]. More recently, in 2018, Lavis et al. identified seven key characteristics of LHS, and categorized LHS as being patient-centred, data- and evidence-driven, system-supported, and enabled through culture and competencies [8]. Despite the ability of LHS to integrate evidence to practice in a cyclical, timely manner, few organizations are classified as LHS. A 2019 report found that only 15% of healthcare leaders described their organization as being able to integrate data into healthcare delivery processes [9]. Additional work aimed at enabling LHS adoption among organizations is urgently needed.

A critical gap in the literature is the lack of evidence related to the implementation of a LHS, specifically in low-income settings or specific healthcare environments, such as hospitals. A systematic review of LHS reported positive impacts, such as shorter patient wait times, reduced postoperative complications, quicker clinical practice decision-making and increased time savings, associated with LHS that were data-driven across multiple countries and sectors [10]. However, none of the included sources were from low-income countries, and specific strategies for implementing LHS were not described [10]. Menear et al. conducted a scoping review in 2019 to develop a framework to support the implementation of LHS [11]. While their findings provide a valuable framework for supporting LHS uptake, the study was limited to a Canadian context and did not identify strategies or interventions commonly used in LHS implementation [11]. Another comprehensive scoping review of 276 papers identified 98 sources specifically reporting on the implementation of LHS [12]. While this paper provided a broad overview of LHS definitions, frameworks and intervention descriptions, the authors did not synthesize the implementation data across studies to highlight effective strategies for implementation or outcome measures [12]. A 2021 review by Allen et al. synthesized 17 LHS frameworks and developed a logic model for assisting future LHS research [13]. While this review provides an excellent starting point for LHS implementation, the identified aspects of the logic model have not yet been mapped to existing LHS [13]. Two additional scoping reviews were identified which provide further details about LHS efforts across clinical contexts [14, 15]. While these reviews contribute to the broader LHS literature, there is a need to synthesize the evidence related to LHS implementation using systematic review methodology. No other reviews have been identified as being relevant to the research topic.

Without a solid understanding of specific strategies and outcome measures used in the implementation of LHS in practice, it is difficult to evaluate the transition of health systems into LHS. Implementation strategies can include any method of facilitating uptake of an action, behaviour or process [16]. There is extensive literature describing different implementation strategies for use in various practice settings or phases of research. The Expert Recommendations for Implementing Change (ERIC) taxonomy is one of the most comprehensive lists of implementation strategies that may be used in interventions [16]. Additionally, Tierney et al. describe 10 key implementation measures to assess in review studies [17]. These measures are valuable for understanding how well an implementation strategy is adopted in practice. Finally, whether an intervention leads to improved patient, provider, population and/or cost-related outcomes is equally important. These outcomes align with the Canadian Institutes of Health Research (CIHR) quadruple aim [18] and are a key focus of emerging health system research [19]. Understanding these three aspects of implementation will provide valuable insight into how a LHS can be achieved.

A mixed-methods review is proposed to identify and synthesize the existing evidence on effective implementation strategies and outcomes of LHS in an international context. This aim will be addressed by answering the following research question: How do healthcare organizations implement and evaluate the transformation of LHS? The findings of this review will be of value to health systems leaders and researchers, as it is anticipated that this insight will provide a structure for healthcare organizations to transition to a LHS. Policy-makers may use these findings to establish health system priorities and pathways to enable LHS.

## Methods

### Study design

A mixed-methods systematic review will be conducted following the Joanna Briggs Institute (JBI) methodology for mixed-methods reviews [20]. Mixed-methods reviews allow for the inclusion of both qualitative and quantitative study designs and are best suited to broad research questions or phenomena of interest [20]. The review has been registered online with PROSPERO (CRD42022293348). To ensure transparency of search results, the Preferred Reporting Items for Systematic Reviews and Meta-Analyses (PRISMA) guidelines will be used to report the mixed-methods review results [21], while the reporting of this protocol paper was guided by the PRISMA extension for review protocols (PRISMA-P) checklist [22].

### Inclusion criteria

Following JBI guidelines for mixed-methods systematic reviews [20], this review will use a PICo (population; phenomenon of interest; context) format for question development and to determine inclusion criteria. This review will aim to answer the following research question: How do healthcare organizations implement and evaluate the transformation of LHS?

#### Population

This review will consider studies that include any mention of LHS, including “rapid learning systems”, “rapid learning health care/healthcare”, “learning health care/healthcare systems” or “learning health systems”. Due to the inconsistent terminology around LHS in the literature [12, 23], only studies utilizing one of these terms will be included. Studies which attempt to describe LHS, or only describe features of a LHS, without using this terminology will be excluded. This approach aligns with previous LHS systematic and scoping reviews, which limit their search terms to “learning health system”, “learning healthcare system” and/or “learning health care system” to reflect the most consistent LHS terminology [11, 12, 14].

#### Phenomenon of interest

This review will consider studies reporting on implementation strategies and outcome measures associated with the adoption of LHS across healthcare settings. Implementation strategies include any procedure or method to implement, assess or evaluate the uptake of LHS in any healthcare setting. The ERIC taxonomy of 73 strategies will be used to determine what qualifies as an implementation strategy [16]. This review will also consider any implementation outcome measures described in studies.

#### Context

This review will consider studies that focus on any healthcare setting. This includes hospitals, academic medical centres, primary care clinics, community health centres, practice-based health networks, healthcare organizations, and individual units or wards that provide inpatient or outpatient services. Any country, size of healthcare organization and other setting characteristics are appropriate for this review. Any non-healthcare settings, such as academic centres and government or nongovernmental organizations where care is not directly provided to patients, will be excluded.

#### Types of studies

This mixed-methods review will consider quantitative, qualitative and mixed-methods study designs for inclusion. Mixed-methods studies will be included if the individual qualitative and quantitative data can be extracted separately. Included studies will be limited to peer-reviewed, published, full-text papers or grey literature sources (e.g. policy reports, conference abstracts) that describe implementation strategies and/or outcome measures of LHS. No restrictions will be placed on language or year of publication. Protocol papers will be excluded, but forward citation searching will be conducted for any published intervention studies stemming from an identified protocol. Similarly, reviews will be excluded but the reference list will be manually searched for relevant papers. The reference lists of all included studies will be manually searched for additional, relevant papers.

### Search strategy

A comprehensive search strategy was developed by a research librarian (MR). Key terms related to LHS, implementation and healthcare organizations formed the basis of an initial limited search of two databases, CINAHL and MEDLINE. After reviewing terms from titles, abstracts and index terms and checking search results against target articles, a final, more open search strategy was developed. An example search strategy for CINAHL can be found in Additional file 1: Appendix 1. The search strategy will be adapted for each chosen database, which will include CINAHL (EBSCOhost), MEDLINE (Ovid), Embase (Elsevier), Nursing & Allied Health Database (ProQuest), PAIS [Public Affairs Information Service] (ProQuest) and Scopus (Elsevier). Boolean operators and relevant controlled vocabulary terms will be used as needed for each database search.

A grey literature search will be conducted to identify additional relevant articles. This will involve searching ProQuest’s Dissertations & Theses Global and reviewing the websites of three pre-identified organizations, namely (1) McMaster Health Forum (https://www.mcmasterforum.org/), (2) Agency for Healthcare Research and Quality (https://www.ahrq.gov/) and (3) The Learning Healthcare Project (https://learninghealthcareproject.org/), and a systematic Google search to identify other relevant materials.

### Study selection

Following a search of all databases, retrieved titles will be uploaded to Covidence data management software (Veritas Health Innovation, Melbourne, Australia). Duplicates will be removed electronically. Three team members (MS, CC, JC) will independently screen all titles and abstracts, making decisions using the predetermined inclusion criteria. Following title and abstract screening, the full texts of relevant papers will be sourced and assessed for inclusion by three independent reviewers (MS, CC, JC), using Covidence. A fourth reviewer will be consulted when conflicts arise between reviewers. Reviewers will discuss the suitability of the paper for inclusion, based on inclusion/exclusion criteria, until a consensus is reached. Papers that do not meet the study criteria will be excluded, with reasons for exclusion reported in the 2020 PRISMA flow diagram [21].

### Data extraction

Following full-text screening, data will be extracted from each included study using a standardized extraction form. Data extraction will be pilot-tested by two reviewers on a subsample of studies, and then completed by one reviewer (MS). Data extraction will be verified by a second reviewer to ensure consistency and reliability of the data. Regular team meetings will be held throughout the data extraction phase to discuss any concerns arising with included studies.

This mixed-methods review will follow a convergent integrated approach, where quantitative and qualitative data are extracted separately and then synthesized [20]. Qualitative data will be extracted as themes and subthemes as related to the research question. Data from qualitative studies will include findings related to LHS and implementation strategies. Evaluation measures and outcome data will also be extracted. This may include a description of how a LHS was implemented or qualitative findings on the LHS impact. Quantitative data will include descriptive or analytical findings related to the implementation and evaluation of LHS. This may include changes in health outcomes, impact on health system, or cost effectiveness. All studies will be extracted for study characteristics including year of publication, country, study design, description of LHS, types of implementation strategy(ies) employed, types of outcome measure(s) used, objective, study setting, population and sample size. An example of the data extraction tools can be found in Additional file 1: Appendix 2. The data extraction tools will be modified as necessary during the data extraction process, as details about included studies are refined. The finalized data extraction tools will be included in the published systematic review.

### Critical appraisal

All studies will undergo critical appraisal using the relevant JBI critical appraisal checklist, based on study design [24]. Two independent reviewers will complete critical appraisal of all studies. The two scores for each study will be averaged and a final score will be reported as a percentage. When significant differences exist between appraisal scores, a third reviewer will be consulted until a consensus about each scoring item has been reached.

### Data synthesis and triangulation

This mixed-methods systematic review will use a convergent integrated approach to data synthesis, where quantitative and qualitative data are first assessed separately, followed by integration of data [20]. First, tables will be used to present the extracted data, categorized based on study design (quantitative, qualitative or mixed-methods). An accompanying overview of all study characteristics will be presented in the text. Next, a narrative synthesis will be provided for each study to describe the key findings and contextual data. This will include reporting similar findings together within each study design category. Implementation outcome data will be categorized using the ERIC taxonomy of 73 implementation strategies [25], Tierney et al.’s list of 10 implementation outcome measures [17] and Allen et al.’s LHS logic model components [13]. Characteristics of LHS will be categorized using Lavis et al.’s list of seven LHS characteristics: (1) patient engagement; (2) digital capture/infrastructure; (3) timely production of research evidence; (4) appropriate decision supports; (5) aligned governance, finance and delivery arrangements; (6) culture of rapid learning and improvement; and (7) competencies for rapid learning and improvement [8].

Following data synthesis, a triangulation protocol will be used to integrate the qualitative and quantitative findings, which will be presented as themes and subthemes. A triangulation protocol is a detailed approach to examine meta-themes across findings from different data sources that have already been analysed individually [26]. We will create a convergence-coding matrix to cross-tabulate the quantitative results with the qualitative themes [27]. To determine common patterns across the data, the review team will meet to discuss the convergence-coding matrix, including separate quantitative and qualitative findings and similarities and differences in outcomes. The resulting integrated data will be presented in a table and as a narrative synthesis.

## Discussion

There is an emerging body of literature describing the LHS concept and its potential impact on value-based healthcare. Despite the growing evidence on what defines a LHS, there are no comprehensive systematic reviews explaining how to implement a LHS in practice. Therefore, a mixed-methods systematic review is proposed to address this gap in research.

A comprehensive search of six electronic databases is proposed to identify papers related to the implementation and evaluation of LHS in an international healthcare context. This review is strengthened by its mixed-methods design and comprehensive search strategy. A rigorous approach to data synthesis will be conducted, following JBI methodology and using the PRISMA guidelines to ensure transparency. The use of the ERIC taxonomy to categorize implementation strategies is another strength of this review. It may not be feasible to conduct a meta-analysis of included studies, which may limit our findings to descriptive results rather than causal factors. Despite this shortcoming, this review will provide important insight into how implementation strategies have been used to transition health systems into LHS, which will inform future intervention studies. Researchers, health system administrators and policy-makers may directly apply the implementation strategies identified in this review to their own healthcare system. The findings can therefore provide an initial roadmap for knowledge users, which will be extremely valuable in implementation of LHS across various health settings and countries.

## Supplementary Information

**Additional file 1: Appendix 1.** Search strategy. **Appendix 2.** Data extraction instruments.

## Data Availability

Data sharing is not applicable to this article as no datasets were generated or analysed during the current study.

## Abbreviations

ERIC: Expert Recommendations for Implementing Change
JBI: Joanna Briggs Institute
LHS: Learning health system(s)
PRISMA: Preferred Reporting Items for Systematic Reviews and Meta-Analyses
