# Supplementary material for: What implementation strategies and outcome measures are used to transform healthcare organizations into learning health systems? A mixed-methods review protocol
Source: Health Res Policy Syst. 2022 Sep 6;20:97. doi: 10.1186/s12961-022-00898-z (PMC9446707; doi:10.1186/s12961-022-00898-z)
Supplement: Supplementary file 1 — Additional file 1: Appendix 1. Search strategy. Appendix 2. Data extraction instruments. [file 12961_2022_898_MOESM1_ESM.docx]

# Appendix 1: Search strategy

The following table presents the search strategy used with the CINAHL (EBSCOhost) and Medline (Ovid) databases. This search was conducted on December 1, with no filters applied.

CINAHL  (EBSCO)

| **Search** | **Query** | **Records retrieved** |
| --- | --- | --- |
| 1 | (MH "Learning Health System") | 19 |
| 2 | TI ("learning health system*" OR "learning health care system*" OR "learning healthcare system*" OR "rapid learning system*" ) OR AB ( "learning health system*" OR "learning health care system*" OR "learning health care system*" OR "rapid learning system*" ) | 479 |
| 3 | 1 or 2 | **484** |
| No limitations included. | |  |

Medline (Ovid)

| **Search** | **Query** | **Records retrieved** |
| --- | --- | --- |
| 1 | Learning Health System/ | 156 |
| 2 | (learning health system* or learning health care system* or learning health care system* or rapid learning system*).ab,kf,kw,ti. | 1008 |
| 3 | 1 or 2 | **1037** |
| No limitations included. | | |

# Appendix 2: Data extraction instruments

**Table 2a**. Qualitative data extraction instrument

| **Reviewer Initials:** | **Date:** | |
| --- | --- | --- |
| **Item** | **Extracted Data** | |
| **First Author (year)** |  | |
| **Title** |  | |
| **Journal/Source** |  | |
| **Study type (e.g. original research)** |  | |
| **Country** |  | |
| **Study objective** |  | |
| **Study design** |  | |
| **Country** |  | |
| **Study setting (e.g. hospital, primary care)** |  | |
| **Resource setting (e.g. low-income, middle-income countries)** |  | |
| **Population (e.g. children, older adults, NA)** |  | |
| **Method of data collection** | - Surveys - Interviews - Focus groups - Other (i.e. Case studies, field notes) | |
| **Description of implementation** |  | |
| **Implementation strategy(ies) used (based on the ERIC taxonomy)** |  |  |
| **Description of learning health system** |  | |
| **Aspect(s) of learning health system included in study** | - Patient engagement - Digital capture/infrastructure - Timely production of evidence - Appropriate decision supports - Aligned governance, finance & deliverables - Culture of rapid learning & improvement - Competencies for rapid learning & improvement | |
| **Implementation measures assessed** | - Acceptability - Adoption - Appropriateness - Feasibility - Fidelity - Implementation cost - Intervention complexity - Penetration - Reach - Sustainability | |
| **Reported impact outcomes** | - Patient-related - Provider-related - Population-related - Cost-related | |
| **Key outcome(s) identified** |  | |
| **Main finding(s) and conclusion described in study** | Theme/sub-theme labels: | |

**Table 2b**. Quantitative data extraction instrument

| **Reviewer Initials:** | **Date:** | |
| --- | --- | --- |
| **Item** | **Extracted Data** | |
| **First Author (year)** |  | |
| **Title** |  | |
| **Journal/Source** |  | |
| **Study type (e.g. original research)** |  | |
| **Country** |  | |
| **Study objective** |  | |
| **Study design** |  | |
| **Country** |  | |
| **Study setting (e.g. hospital, primary care)** |  | |
| **Resource setting (e.g. low-income, middle-income countries)** |  | |
| **Population (e.g. children, older adults, NA)** |  | |
| **Method of data collection** | - Surveys - Health admin data - Other (i.e. Financial data) | |
| **Description of implementation** |  | |
| **Implementation strategy(ies) used (based on the ERIC taxonomy)** |  |  |
| **Description of learning health system** |  | |
| **Aspect(s) of learning health system included in study** | - Patient engagement - Digital capture/infrastructure - Timely production of evidence - Appropriate decision supports - Aligned governance, finance & deliverables - Culture of rapid learning & improvement - Competencies for rapid learning & improvement | |
| **Implementation measures assessed** | - Acceptability - Adoption - Appropriateness - Feasibility - Fidelity - Implementation cost - Intervention complexity - Penetration - Reach - Sustainability | |
| **Reported impact outcomes** | - Patient-related - Provider-related - Population-related - Cost-related | |
| **Key outcome(s) identified** |  | |
| **Main finding(s) and conclusion described in study** |  | |
